# External Validation of the A^2^DS^2^ Score to Predict Stroke-Associated Pneumonia in a Chinese Population: A Prospective Cohort Study

**DOI:** 10.1371/journal.pone.0109665

**Published:** 2014-10-09

**Authors:** Yapeng Li, Bo Song, Hui Fang, Yuan Gao, Lu Zhao, Yuming Xu

**Affiliations:** Department of Neurology, the First Affiliated Hospital of Zhengzhou University, Zhengzhou, Henan Province, China; Massachusetts General Hospital, United States of America

## Abstract

**Background and Purpose:**

The A^2^DS^2^ score was recently developed from the Berlin Stroke Registry for predicting in-hospital pneumonia after acute ischemic stroke and performed well in an external validation in the North-west Germany Stroke Registry. It could be a useful tool for risk stratification in clinical practice or stroke trials. We aimed to prospectively validate the predictive value of A^2^DS^2^ score in a Chinese stroke population.

**Methods:**

The prognostic model was used to predict stroke-associated pneumonia (SAP) from Henan Province Stroke Registry (HNSR) in which data were prospectively collected. The receiver-operating characteristic curves were plotted, and the C statistics were calculated to assess the discrimination ability. The Hosmer–Lemeshow goodness-of-fit test and the plot of observed versus predicted SAP risk were used to assess model calibration.

**Results:**

Among 1142 eligible patients, the overall in-hospital SAP was 18.8%, which ranged from 9.0% in patients with lower A^2^DS^2^ scores (0–4) to 65.0% in those with higher scores of 5 to 10 (P for trend <0.001). The C statistic was 0.836 (95% confidence interval, 0.803–0.868) through the A^2^DS^2^ score, suggesting excellent discrimination in the HNSR. The A^2^DS^2^ score also showed excellent calibration (Cox and Snell *R* ^2^ = 0.243) in the external validation sample from the HNSR.

**Conclusions:**

The A^2^DS^2^ score could reliably predict in-hospital SAP in Chinese stroke patients. It might be helpful for the assessment of increased risk monitoring and prophylactic treatment in identified high-risk patients for SAP in clinical routine.

## Introduction

Pneumonia is a major cause of in-hospital morbidity and mortality in acute stroke population [1]–[11], and attributes to increase the length of hospital stay and hospitalization cost [12]–[16].

Previous studies have identified several factors being independently associated with stroke-associated pneumonia (SAP) in acute stroke, such as older age, stroke severity, dysphagia, impaired level of consciousness, diabetes mellitus, location of stroke infarction and so on [11], [12], [16]–[27]. Some of these findings have been translated into scoring systems aiming at risk stratification for SAP [19]–[24]. However, no reliable scoring system is currently available in routine clinical practice or stroke trials.

The A^2^DS^2^ score (Table S1) was recently developed from the Berlin Stroke registry (BSR) cohort to assess the risk of in-hospital SAP based on routinely collected data and externally validated its predictive properties in the independent North-west Germany Stroke Registry(NGSR) [22]. It may be a useful tool for clinical practice and stroke trials. We aimed to prospectively validate the A^2^DS^2^ score in a Chinese stroke population.

## Methods

### Patients selection

Patients included in this study were from the database of the Henan Province Stroke Registry (HNSR), which is a prospective single-center hospital–based cohort study of consecutive patients who had transient ischemic attack (TIA) and acute ischemic stroke (stroke onset to hospital ≤7 days) [28].

Detailed baseline data were registered prospectively using standardized case report forms. Acute ischemic stroke registry forms were completed by neurologists with similar levels of training and experience. To be eligible for this study, subjects had to meet the following criteria: (1) age ≥18 years; (2) hospitalized with a primary diagnosis of acute ischemic stroke according to World Health Organization criteria [29]; (3) stroke confirmed by CT or MRI. Patients were excluded if any of the components of the A^2^DS^2^ score were not available.

### Ethics Statement

The study was approved by the central Institutional Review Board at the first affiliated hospital of Zhengzhou university. All patients or their designated relatives were informed about study participation, and informed written consent was obtained.

### Data definitions

The following variables were analyzed for the present study: (1)demographics (age and sex); (2)stroke risk factors: hypertension (history of hypertension or antihypertensive medication use), diabetes mellitus (history of diabetes mellitus or antidiabetic medication use), dyslipidemia (history of dyslipidemia or lipid-lowering medication use), atrial fibrillation (history of atrial fibrillation or documentation of atrial fibrillation at admission), coronary heart disease, history of stroke and TIA, current smoking, and excess alcohol consumption (≥2 standard alcohol beverages per day); (3)admission stroke severity based on National Institutes of Health Stroke Scale score (NIHSS); (4)clinical feature: dysphagia (symptom of dysphagia, or abnormal of swallowing water test, or decline of consciousness level, or cough asthenia); (5)stroke subtype: according to the Oxfordshire Community Stroke Project criteria (OCSP) [30]; (6)complication: pneumonia; (7)length of hospital stay.

In this study, SAP was diagnosed by treating physician according to the Centers for Disease Control and Prevention criteria for hospital-acquired pneumonia [31], on a basis of clinical and laboratory indices of respiratory tract infection (fever, cough, auscultatory respiratory crackles, new purulent sputum, or positive sputum culture), and supported by typical chest X-ray or CT findings. Only hospital-acquired pneumonia was documented and pneumonia before stroke was not considered. Data on in-hospital SAP was prospectively recorded.

### Outcomes

The main outcome of interest was in-hospital SAP.

### Statistical analysis

The Shapiro-Wilk test was used to check the normality of continuous variables. Student *t* test was used in the case of normality, and Mann-whitney test was used in the case of abnormality. The differences between categorical variables were analyzed by χ^2^ test. Discrimination was assessed by calculating the area under the receiver operating characteristic curve (AUROC). Calibration was assessed by performing the Hosmer–Lemeshow goodness of fit test and was graphically depicted in the plot of observed versus predicted SAP risk according to 7 deciles of predicted risk. Univariate and multivariable logistic regression was performed to determine the independent predictors of SAP after acute ischemic stroke. Variables associated with SAP at a significance level of 0.1 in univariate analysis were included in a multivariable logistic regression model using a stepwise backward elimination procedure.

All tests were 2-tailed and statistical significance was determined at an α level of 0.05. Statistical analyses were performed with the SPSS 16.0.

## Results

### Patients flow

**Figure 1:**
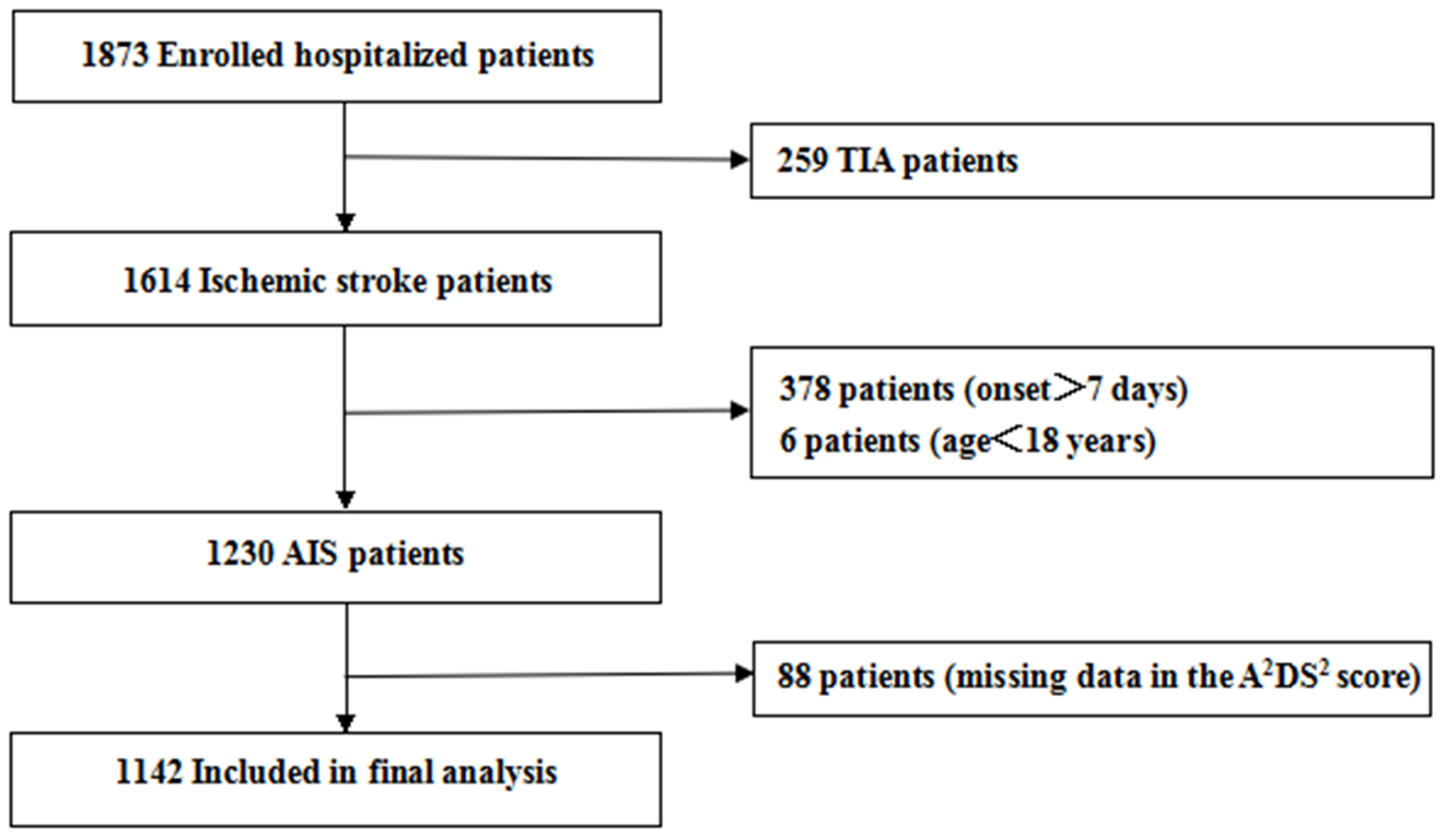
Flowchart of study population. The flowchart was used to illustrate how the study population was selected.

From Jan 2009 to Dec 2012, a total of 1873 patients of acute ischemic stroke were registered in the HNSR cohort. Of these, we excluded 259 patients with TIA, 378 patients with onset>7 days, 6 patients with age <18 years, and an additional 88 patients who had missing data of ≥1 components in the A^2^DS^2^ score. Finally, 1142 eligible patients were included in our analysis (Figure 1).

### Baseline characteristics

The baseline characteristics of the HNSR and BSR cohort were summarized in the Table 1. The patients in HNSR were quite different from those in BSR in demographic characteristics and risk factors. The Chinese patients were younger, had a higher proportion of men, presented somewhat milder severity, and lower incidence of atrial fibrillation. Among the 1142 eligible patients, the overall in-hospital SAP was 18.8%, which increased from 9.0% in patients with lower A^2^DS^2^ scores (0–4) to 65.0% in those with higher scores of 5 to 10 (P for trend <0.001).

**Table 1:** Comparison of Patient Baseline Characteristics Between the HNSR and BSR Cohort Which Was Initially Used to Introduce the A^2^DS^2^ Score.

Characteristic	HNSR Cohort	BSR Cohort
	(n = 1142)	(n = 15335)
Age, y		
Mean(SD)	60.3±13.1	71.2±13.1
Median(IQR)	61(51–70)	72(64–81)
Age group, no.(%), y		
≤64	692(60.6)	4006(26.1)
65–74	285(25.0)	4659(30.6)
75–84	140(12.3)	4311(28.1)
≥85	25(2.2)	2323(15.1)
Male sex, no.(%)	723(63.3)	7759(50.6)
Stroke severity, NIHSS		
Median(IQR)	4(1–7)	4(2–10)
NIHSS categories, no.(%)		
0–4	654(57.3)	6290(50.4)
5–15	420(36.8)	5098(37.1)
16+	68(6.0)	1707(12.4)
Dysphagia, no.(%)	217(19.0)	3505(22.9)
Comorbidities, no.(%)		
Hypertension	673(58.9)	11358(85.9)
Diabetes mellitus	267(23.4)	4783(31.2)
Atrial fibrillation	51(4.5)	4139(27.0)
Previous stroke	299(26.2)	4320(28.2)
Length of stay, d		
Mean(SD)	15.7(9.3)	9.2(7.3)
Median(IQR)	14.0(10–18)	8.0(5–11)
Pneumonia, no.(%)	215(18.8%)	7.2%

HNSR, Henan Province Stroke Registry; BSR, Berlin Stroke Registry; SD, standard deviation; IQR, interquartile range; NIHSS, National Institutes of Health Stroke Scale.

### Discrimination and calibration

**Figure 2:**
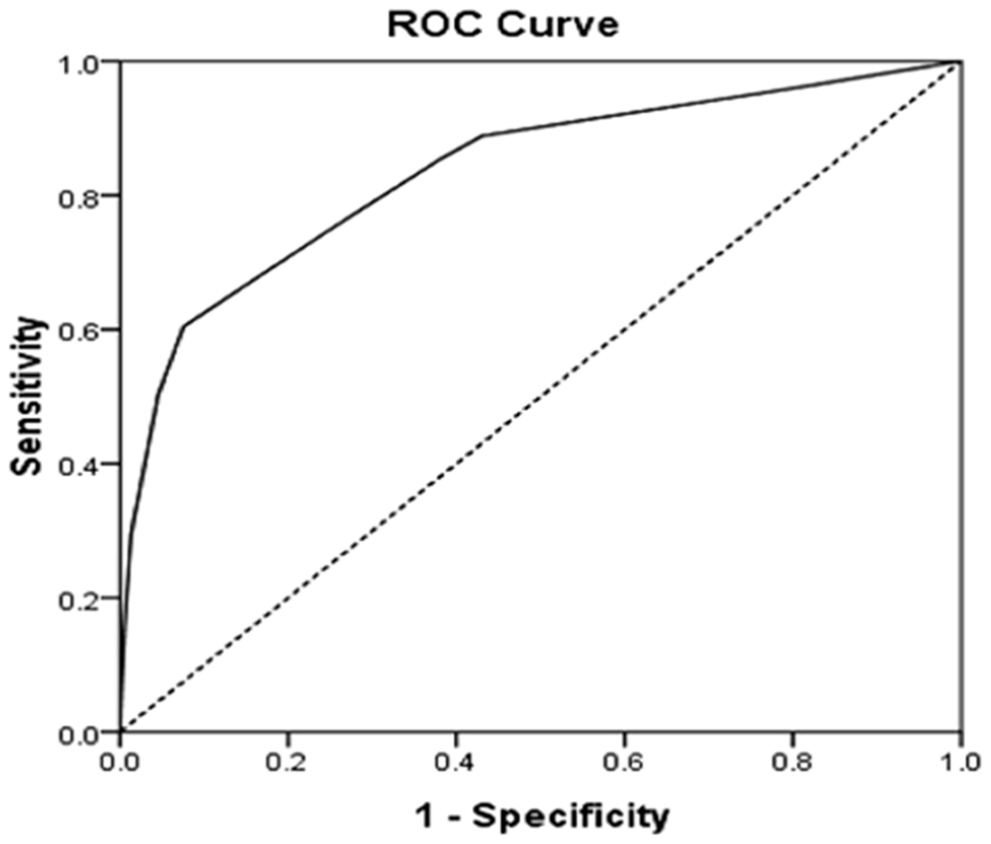
Receiver-operator curve of the A^2^DS^2^ score in the HNSR cohort. The A^2^DS^2^ score showed excellent discrimination with an area under the receiver operating characteristic curve of 0.836 (95% confidence interval, 0.803–0.868).

The A^2^DS^2^ score showed excellent discrimination (C statistic: 0.836, 95% confidence interval: 0.803–0.868, P<0.001) (Figure 2).

The sensitivity, specificity, positive predictive value, negative predictive value and Youden index for each individual point were listed in Table 2. Acute ischemic stroke patients were divided into low A^2^DS^2^ group (0–4) and high A^2^DS^2^ group (5–10) based on optimal cut-off point which represents maximum Youden index.

**Table 2:** Discrimination abilities of each point about the A^2^DS^2^ score.

A^2^DS^2^ score	sensitivity	specificity	PPV	NPV	Youden index
0	0.1000	0.0000	0.188	NA	0
1	0.9628	0.1855	0.215	0.956	0.1483
2	0.8884	0.5696	0.324	0.957	0.4580
3	0.8558	0.6170	0.341	0.949	0.4728
4	0.7442	0.7562	0.415	0.927	0.5004
5	0.6047	0.9245	0.650	0.910	0.5292*
6	0.5023	0.9547	0.720	0.892	0.4570
7	0.2930	0.9871	0.840	0.858	0.2801
8	0.1581	0.9946	0.872	0.836	0.1527
9	0.0419	0.9989	0.900	0.818	0.0408

PPV, positive predictive value; NPV, negative predictive value; NA, not applicable.

*maximum Youden index.

**Figure 3:**
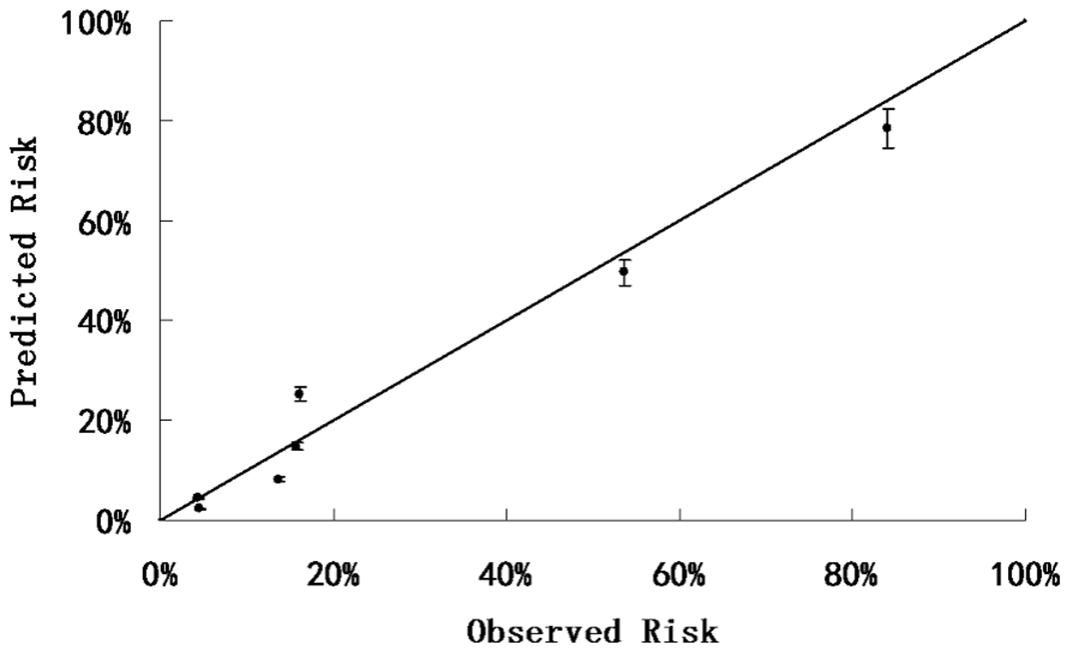
The plot of observed versus predicted risk of SAP in the HNSR cohort. There was a high correlation between observed and expected probability of SAP (Pearson correlation coefficient, 0.987).

The Hosmer–Lemeshow goodness of fit test showed that the A^2^DS^2^ score also had an excellent calibration (Cox and Snell *R* ^2^ = 0.243) (Table 3). The plot of observed versus predicted risk of in-hospital SAP after acute ischemic stroke showed high correlation (Pearson correlation coefficient: 0.987) between observed and predicted risk in the external validation cohort (Figure 3).

**Table 3:** Discrimination and calibration of the A^2^DS^2^ score in different cohort.

	BSR Cohort	NGSR Cohort	HNSR Cohort
Discrimination			
AUROC(95%CI)	0.837(0.826–0.849)	0.835(0.828–0.842)	0.836(0.803–0.868)
Calibration, goodness of fit			
Cox and Snell *R* ^2^	0.106	0.112	0.243
Nagelkerke *R* ^2^	0.259	0.264	0.392

BSR, Berlin Stroke Registry; NGSR, North-west Germany Stroke Registry; HNSR, Henan Province Stroke Registry; AUROC, area under the receiver operating characteristic curve.

### Univariate and multivariable logistic regression analysis

The univariate logistic regression analysis results of the risk factors were shown in the Table 4. Age, atrial fibrillation, dysphagia, admission NIHSS score, history of stroke or TIA, diabetes mellitus, OCSP subtype and length of stay were associated with SAP at the significance level of 0.1. The A^2^DS^2^ score together with other significant variables were included in the multivariable logistic regression analysis using a stepwise backward elimination procedure. The multivariate logistic regression analysis demonstrated that length of stay, history of stroke and the A^2^DS^2^ score were associated with a higher risk of SAP (Table 5).

**Table 4:** Risk factors for SAP by univariate logistic regression.

Characteristics	N	Pneumonia	OR(95%CI)	P Value
Age, y				
<75	977	16.3%	1.0(reference)	
75+	165	33.9%	2.64(1.84–3.81)	0.001*
Male sex	723	18.7%	0.97(0.72–1.32)	0.861
Dysphagia	217	67.3%	25.51(17.53–37.12)	0.001*
Comorbidities				
Atrial fibrillation	51	49.0%	4.56(2.58–8.07)	0.001*
Hypertesion	673	18.9%	1.01(0.75–1.36)	0.964
Diabetes mellitus	343	23.9%	1.57(1.15–2.15)	0.004*
Previous stroke	299	24.7%	1.64(1.19–2.25)	0.002*
Previous TIA	53	28.3%	1.76(0.95–3.25)	0.071*
Dyslipidemia	94	14.9%	0.74(0.41–1.33)	0.309
Coronary heart disease	115	19.1%	1.02(0.63–1.67)	0.930
Excess alcohol consumption	270	16.7%	0.83(0.58–1.19)	0.299
Current smoking	336	17.3	0.86(0.62–1.20)	0.382
NIHSS on admission				
0–4	654	7.8%	1.0(reference)	
5–15	420	27.4%	4.46(3.12–6.37)	0.001*
16+	68	72.1%	30.49(16.70–55.67)	0.001*
OCSP subtype				
LACI	222	12.2%	1.0(reference)	
PACI	403	19.4%	1.73(1.08–2.78)	0.021*
TACI	40	37.5%	4.33(2.03–9.23)	0.001*
POCI	256	23.0%	2.16(1.32–3.55)	0.002*
Length of stay	921	20.4%	1.10(1.08–1.12)	0.001*

OR, Odds Ratio; CI, Confidence Interval; TIA, Transient Ischemic Attack; NIHSS, National Institutes of Health Stroke Scale score; OCSP, Oxfordshire Community Stroke Project; LACI, Lacunar infarction; PACI, Partial anterior circulation infarct; TACI, Total anterior circulation infarct; POCI, Posterior circulation infarct.

**Table 5:** Risk factors for SAP by multivariate logistic regression.

Risk factor	OR	95%CI	P Value
Length of stay	1.06	1.04–1.09	0.001
History of stroke	1.64	1.03–2.60	0.037
The A^2^DS^2^ score	1.76	1.58–1.95	0.001

OR, Odds Ratio; CI, Confidence Interval.

## Discussion

Our results showed that the A^2^DS^2^ score has an excellent discrimination (C statistic: 0.836) and calibration (Cox and Snell *R* ^2^ = 0.243) of predicting the risk of SAP in the HNSR cohort, even if the Chinese patients were significantly different in clinical baseline from those in the Germany (mainly with younger median age, milder stroke severity and lower incidence of atrial fibrillation). This is a prospective external validation study for the A^2^DS^2^ score through a different independent population from the BSR cohort.

The A^2^DS^2^ score was developed based on routinely collected data available directly after hospital admission within a large stroke register in Berlin, and showed good discrimination and calibration abilities both in different population (Germany and Chinese). No reliable scoring system was available in routine clinical practice or stroke research before the A^2^DS^2^ score. The A^2^DS^2^ score could be used to identify patients who are at high risk of developing SAP after stroke, which is important for clinical practice and stroke trials, especially in the study of early prophylactic antibiotics [32], [33].

In the present study, the incidence of in-hospital SAP (18.8%) was significantly higher than the BSR cohort (7.2%). Length of stay may play an important role. The HNSR cohort had longer hospital stay than the BSR cohort (15.7 VS 9.2 days). After adjusting for potential confounders, length of stay was significantly associated with development of SAP (adjusted OR, 1.06; 95% CI, 1.04–1.09; P<0.001). Previous studies also reported that length of stay was highly associated with SAP [13], [16], along with the increasing risk of opportunistic infection. Furthermore, the conventional ward in our department (not stroke unit) may increase the risk of cross-infection with the frequent visit of family.

There were also some limitations in this study. Firstly, this is a single center study that could have selected bias. Secondly, the exact date of new-onset SAP during hospitalization wasn't documented, the present study just showed that the length of stay was highly associated with SAP, but could not come to a conclusion as to whether patients with a longer length of stay was more likely to develop pneumonia or whether diagnosis of pneumonia led to a longer hospitalization. Therefore, whether the A^2^DS^2^ risk model can apply to any stroke care institution needs further investigation.

## Conclusions

In conclusion, our study prospectively validates the predictive accuracy of the A^2^DS^2^ score in Chinese patients by identifying the in-hospital SAP risk of acute ischemic stroke. It might be helpful for the assessment of increased risk monitoring and prophylactic treatment in identified high-risk patients for SAP in clinical routine and stroke trials.

## Supporting Information

Table S1 **Components of the A^2^DS^2^ score.**
(DOC)

Supporting Information S1 **Raw data of the present study.**
(XLS)

## References

[1] Koennecke HC, Belz W, Berfelde D, Endres M, Fitzek S, et al (2011) Factors influencing in-hospital mortality and morbidity in patients treated on a stroke unit. Neurology 77: 965–972. 21865573 10.1212/WNL.0b013e31822dc795

[2] Emsley HC, Hopkins SJ (2008) Acute ischaemic stroke and infection: recent and emerging concepts. Lancet neurology 7: 341–353. 18339349 10.1016/S1474-4422(08)70061-9

[3] Heuschmann PU, Kolominsky-Rabas PL, Misselwitz B, Hermanek P, Leffmann C, et al (2004) Predictors of in-hospital mortality and attributable risks of death after ischemic stroke: the German Stroke Registers Study Group. Archives of internal medicine 164: 1761–1768. 15364669 10.1001/archinte.164.16.1761

[4] Westendorp WF, Nederkoorn PJ, Vermeij JD, Dijkgraaf MG, van de Beek D (2011) Post-stroke infection: a systematic review and meta-analysis. BMC neurology 11: 110. 21933425 10.1186/1471-2377-11-110 PMC3185266

[5] Pandian JD, Kaur A, Jyotsna R, Sylaja PN, Vijaya P, et al (2012) Complications in acute stroke in India (CAST-I): a multicenter study. Journal of stroke and cerebrovascular diseases: the official journal of National Stroke Association 21: 695–703. 21511495 10.1016/j.jstrokecerebrovasdis.2011.03.003

[6] Forti P, Maioli F, Procaccianti G, Nativio V, Lega MV, et al (2013) Independent predictors of ischemic stroke in the elderly: prospective data from a stroke unit. Neurology 80: 29–38. 23243075 10.1212/WNL.0b013e31827b1a41

[7] Grube MM, Koennecke HC, Walter G, Meisel A, Sobesky J, et al (2013) Influence of acute complications on outcome 3 months after ischemic stroke. PloS one 8: e75719. 24086621 10.1371/journal.pone.0075719 PMC3782455

[8] Hong KS, Kang DW, Koo JS, Yu KH, Han MK, et al (2008) Impact of neurological and medical complications on 3-month outcomes in acute ischaemic stroke. European journal of neurology: the official journal of the European Federation of Neurological Societies 15: 1324–1331. 10.1111/j.1468-1331.2008.02310.x 19049549

[9] Tong X, Kuklina EV, Gillespie C, George MG (2010) Medical complications among hospitalizations for ischemic stroke in the United States from 1998 to 2007. Stroke; a journal of cerebral circulation 41: 980–986. 10.1161/STROKEAHA.110.578674 20203317

[10] Kumar S, Selim MH, Caplan LR (2010) Medical complications after stroke. Lancet neurology 9: 105–118. 20083041 10.1016/S1474-4422(09)70266-2

[11] Hilker R, Poetter C, Findeisen N, Sobesky J, Jacobs A, et al (2003) Nosocomial pneumonia after acute stroke: implications for neurological intensive care medicine. Stroke; a journal of cerebral circulation 34: 975–981. 10.1161/01.STR.0000063373.70993.CD 12637700

[12] Kammersgaard LP, Jorgensen HS, Reith J, Nakayama H, Houth JG, et al (2001) Early infection and prognosis after acute stroke: the Copenhagen Stroke Study. Journal of stroke and cerebrovascular diseases: the official journal of National Stroke Association 10: 217–221. 17903827 10.1053/jscd.2001.30366

[13] Ingeman A, Andersen G, Hundborg HH, Svendsen ML, Johnsen SP (2011) In-hospital medical complications, length of stay, and mortality among stroke unit patients. Stroke; a journal of cerebral circulation 42: 3214–3218. 10.1161/STROKEAHA.110.610881 21868737

[14] Katzan IL, Dawson NV, Thomas CL, Votruba ME, Cebul RD (2007) The cost of pneumonia after acute stroke. Neurology 68: 1938–1943. 17536051 10.1212/01.wnl.0000263187.08969.45

[15] Wilson RD (2012) Mortality and cost of pneumonia after stroke for different risk groups. Journal of stroke and cerebrovascular diseases: the official journal of National Stroke Association 21: 61–67. 22225864 10.1016/j.jstrokecerebrovasdis.2010.05.002 PMC3255072

[16] Finlayson O, Kapral M, Hall R, Asllani E, Selchen D, et al (2011) Risk factors, inpatient care, and outcomes of pneumonia after ischemic stroke. Neurology 77: 1338–1345. 21940613 10.1212/WNL.0b013e31823152b1

[17] Chumbler NR, Williams LS, Wells CK, Lo AC, Nadeau S, et al (2010) Derivation and validation of a clinical system for predicting pneumonia in acute stroke. Neuroepidemiology 34: 193–199. 20197702 10.1159/000289350 PMC2883837

[18] Sellars C, Bowie L, Bagg J, Sweeney MP, Miller H, et al (2007) Risk factors for chest infection in acute stroke: a prospective cohort study. Stroke; a journal of cerebral circulation 38: 2284–2291. 10.1161/STROKEAHA.106.478156 17569875

[19] Harms H, Grittner U, Droge H, Meisel A (2013) Predicting post-stroke pneumonia: the PANTHERIS score. Acta neurologica Scandinavica 128: 178–184. 23461541 10.1111/ane.12095

[20] Katzan IL, Cebul RD, Husak SH, Dawson NV, Baker DW (2003) The effect of pneumonia on mortality among patients hospitalized for acute stroke. Neurology 60: 620–625. 12601102 10.1212/01.wnl.0000046586.38284.60

[21] Aslanyan S, Weir CJ, Diener HC, Kaste M, Lees KR (2004) Pneumonia and urinary tract infection after acute ischaemic stroke: a tertiary analysis of the GAIN International trial. European journal of neurology: the official journal of the European Federation of Neurological Societies 11: 49–53. 10.1046/j.1468-1331.2003.00749.x 14692888

[22] Hoffmann S, Malzahn U, Harms H, Koennecke HC, Berger K, et al (2012) Development of a clinical score (A2DS2) to predict pneumonia in acute ischemic stroke. Stroke; a journal of cerebral circulation 43: 2617–2623. 10.1161/STROKEAHA.112.653055 22798325

[23] Ji R, Shen H, Pan Y, Wang P, Liu G, et al (2013) Novel risk score to predict pneumonia after acute ischemic stroke. Stroke; a journal of cerebral circulation 44: 1303–1309. 10.1161/STROKEAHA.111.000598 23482598

[24] Kwon HM, Jeong SW, Lee SH, Yoon BW (2006) The pneumonia score: a simple grading scale for prediction of pneumonia after acute stroke. American journal of infection control 34: 64–68. 16490608 10.1016/j.ajic.2005.06.011

[25] Galovic M, Leisi N, Muller M, Weber J, Abela E, et al (2013) Lesion location predicts transient and extended risk of aspiration after supratentorial ischemic stroke. Stroke; a journal of cerebral circulation 44: 2760–2767. 10.1161/STROKEAHA.113.001690 23887840

[26] Walter U, Knoblich R, Steinhagen V, Donat M, Benecke R, et al (2007) Predictors of pneumonia in acute stroke patients admitted to a neurological intensive care unit. Journal of neurology 254: 1323–1329. 17361338 10.1007/s00415-007-0520-0

[27] Kemmling A, Lev MH, Payabvash S, Betensky RA, Qian J, et al (2013) Hospital acquired pneumonia is linked to right hemispheric peri-insular stroke. PloS one 8: e71141. 23951094 10.1371/journal.pone.0071141 PMC3737185

[28] Song B, Fang H, Zhao L, Gao Y, Tan S, et al (2013) Validation of the ABCD3-I score to predict stroke risk after transient ischemic attack. Stroke; a journal of cerebral circulation 44: 1244–1248. 10.1161/STROKEAHA.113.000969 23532014

[29] Stroke—1989. Recommendations on stroke prevention, diagnosis, and therapy. Report of the WHO Task Force on Stroke and other Cerebrovascular Disorders. Stroke; a journal of cerebral circulation 20: 1407–1431. 10.1161/01.str.20.10.1407 2799873

[30] Bamford J, Sandercock P, Dennis M, Burn J, Warlow C (1991) Classification and natural history of clinically identifiable subtypes of cerebral infarction. Lancet 337: 1521–1526. 1675378 10.1016/0140-6736(91)93206-o

[31] Garner JS, Jarvis WR, Emori TG, Horan TC, Hughes JM (1988) CDC definitions for nosocomial infections, 1988. American journal of infection control 16: 128–140. 2841893 10.1016/0196-6553(88)90053-3

[32] Westendorp WF, Vermeij JD, Vermeij F, Den Hertog HM, Dippel DW, et al (2012) Antibiotic therapy for preventing infections in patients with acute stroke. The Cochrane database of systematic reviews 1: CD008530. 22258987 10.1002/14651858.CD008530.pub2

[33] Harms H, Prass K, Meisel C, Klehmet J, Rogge W, et al (2008) Preventive antibacterial therapy in acute ischemic stroke: a randomized controlled trial. PloS one 3: e2158. 18478129 10.1371/journal.pone.0002158 PMC2373885

