# Supplementary material for: External Validation of the A2DS2 Score to Predict Stroke-Associated Pneumonia in a Chinese Population: A Prospective Cohort Study
Source: PLoS One. 2014 Oct 9;9(10):e109665. doi: 10.1371/journal.pone.0109665 (PMC4192306; doi:10.1371/journal.pone.0109665)
Supplement: Table S1 — Components of the A2DS2 score. (DOC) [file pone.0109665.s001.doc]

**Table S1. Components of the A^2^DS^2^ score.**

| Clinical Variables | Points |
| --- | --- |
| Age  < 75 y  ≥ 75 y  Atrial fibrillation  Dysphagia  Sex  female  Male  Stroke severity (NIHSS)  0-4  5-15  > 16 | 0  1  1  2  0  1  0  3  5 |

NIHSS, National Institutes of Health Stroke Scale score.
